# Cutoff Point of Mini-Balance Evaluation Systems Test Scores for Elderly Estimated by Center of Pressure Measurements by Linear Regression and Decision Tree Classification

**DOI:** 10.3390/life12122133

**Published:** 2022-12-17

**Authors:** Wen-Yen Liao, Yu-Hsiu Chu, Fan-Yu Liu, Kang-Ming Chang, Li-Wei Chou

**Affiliations:** 1 Department of Physical Medicine and Rehabilitation, China Medical University Hospital, Taichung 404332, Taiwan; 2 Department of Physical Therapy and Graduate Institute of Rehabilitation Science, China Medical University, Taichung 406040, Taiwan; 3 Department of Computer Science and Information Engineering, Asia University, Taichung 413505, Taiwan; 4 Department of Digital Media Design, Asia University, Taichung 413505, Taiwan; 5 Department of Medical Research, China Medical University Hospital, China Medical University, Taichung 406040, Taiwan; 6 Department of Physical Medicine and Rehabilitation, Asia University Hospital, Asia University, Taichung 413505, Taiwan

**Keywords:** aging, Balance Evaluation Systems Test, center of pressure, decision tree, fall, linear regression, rehabilitation

## Abstract

Background: Understanding balance ability and assessing the risk of possible falls are very important for elderly rehabilitation. The Mini-Balanced Evaluation System Test (Mini-BESTest) is an important survey for older adults to evaluate subject balance, but it is not easy to complete due to various limitations of physical activities, including occasional fear of injury. A center of pressure (CoP) signal can be extracted from a force pressure plate with a short recording time, and it is relatively achievable to ask subjects to stand on a force pressure plate in a clinical environment. The goal of this study is to estimate the cutoff score of Mini-BESTest scores from CoP data. Methods: CoP signals from a human balance evaluation database with data from 75 people were used. Time domain, frequency domain, and nonlinear domain parameters of 60 s CoP signals were extracted to classify different cutoff point scores for both linear regression and a decision tree algorithm. Classification performances were evaluated by accuracy and area under a receiver operating characteristic curve. Results: The correlation coefficient between real and estimated Mini-BESTest scores by linear regression is 0.16. Instead of linear regression, binary classification accuracy above or below a cutoff point score was developed to examine the CoP classification performance for Mini-BESTest scores. The decision tree algorithm is superior to regression analysis among scores from 16 to 20. The highest area under the curve is 0.76 at a cutoff point score of 21 for the CoP measurement condition of eyes opened on the foam, and the corresponding classification accuracy is 76.15%. Conclusions: CoP measurement is a potential tool to estimate corresponding balance and fall survey scores for elderly rehabilitation and is useful for clinical users.

## 1. Introduction

In recent years, aging populations have become a topic of public concern, and many countries have begun transitioning to aging societies one after another. Therefore, healthcare for the elderly is an important issue. The fall and balance ability of the elderly are important factors affecting the quality of life of the elderly. Because balance ability is an ability to maintain a stable state of the body, with the increase of age from young to old, balance ability will also become stronger with age, until the balance ability reaches fullness in middle age, and then old age begins to degrade balance ability due to physical decline. When the elderly fall, it will cause the loss of independent living ability and impact their quality of life. Therefore, it is very important to understand the balance ability of the elderly in advance and assess the risk of possible falls. The Balance Evaluation Systems Test (BESTest) is used to evaluate a person’s degree of poor posture balance. According to the BESTest official website profile, the BESTest differentiates balance into six basic systems that may limit balance: biomechanical constraints, stability limits, anticipatory postural adjustments, reactive postural response, sensory orientation, and stability in gait; these are derived from a 27-question questionnaire [1]. This unique assessment tool for patients with Parkinson’s disease, neurological disease, head injury, stroke, cerebral palsy, cognitive impairment, or other balance disorders can help, through the identification of subtle deficits and changes in treatment, provide the treatment the patient needs. The short version of the BESTest, the Mini-Balance Evaluation Systems Test (Mini-BESTest), consists of 14 items categorized into four distinct domains of human balance: anticipatory postural adjustment, reactive postural response, sensory orientation, and dynamic gait [2]. According to the study of Abigail L. Ledd et al., 80 patients with Parkinson’s disease used the BESTest and the Mini-BESTest to evaluate their balance utility. The Mini-BESTest has fewer items and takes only 15 min to complete, but it is as reliable as BESTest and has better discriminative performance in identifying Parkinson’s disease patients.

One of the physiological signals to detect the balance mechanism of the human body is the center of pressure (CoP) of the foot, and the measurement of the CoP is performed by a biomechanical force plate. When the body’s sense of balance changes, the parallel force also changes. The force plate will collect force plate moment data along the front-to-back direction, the left–right direction, and the up–down direction. These CoPs can be used to calculate the position of the center of pressure relative to the origin of the force plate. There have been many studies using CoP measurements to detect body balance and falls [3]. Fall detection is a common application of CoPs. According to a 2019 review article, sway area per unit time, anteroposterior mean velocity, and radial mean velocity were the best traditional features to distinguish fall and no-fall subjects [4]. Da Conceição et al. also used CoP parameters to estimate the probability of falling in Parkinson’s disease patients over the next 4-12 months [5]. Estévez-Pedraza studied the balance index of CoP parameters, compared with the results of a Modified Wii Balance Board, and used multiple logistic regression models to test the association of 78 CoP parameters with the Wii Balance Board [6]. The CoP recording durations ranged from 20 s to 60 s.

Compared to CoP recording, the equipment for the Mini-BESTest is cheaper and generally easier to use. However, if the subjects are unwilling to match due to various personal factors, there is no way to use them. Using other measurements to estimate the questionnaire is a good approach to solve such a problem. For example, Romaniszyn et al. estimated Berg Balance Scale scores by video [7]. Aldenhoven et al. also investigated elder imbalance via a camera-based approach; compared with the Romberg test, the system achieved a sensitivity of 80% and a specificity of 87% [8].The other novel approach is using CoPs to estimate the questionnaire. In [9], the author uses CoP feature parameters and AI algorithms to estimate the Falls Efficacy Scale from young to old and achieves good results. Therefore, using CoPs to estimate the questionnaire is a potential approach. However, the Mini-BESTest score is an important tool for clinicians to evaluate subject status. If CoP data is used to evaluate the Mini-BESTest score, the most traditional way is the regression model [10,11]. By entering the CoP parameters as inputs, the regression model estimates the parameters with statistical effects and also estimates the Mini-BESTest score from these statistically significant input features. If the actual Mini-BESTest score is very close to the score estimated by the regression method, the correlation coefficient between the two scores can also be used. Generally speaking, a correlation coefficient >0.7 is a high correlation, one between 0.3–0.7 is a moderate correlation, and one <0.3 is a low correlation. When the two variables are lowly correlated, there are large errors between estimated Mini-BESTest scores and real Mini-BESTest scores. A binary classifier is used to evaluate whether the estimated score is above or below the cutoff score. The Mini-BESTest cutoff score is highly related to clinical problems, so it is clinically practical to evaluate the classification accuracy of the cutoff score. Artificial intelligence has been widely used in binary classification, and a decision tree algorithm (DT) is one of the methods for predicting the test group by classifying the characteristics of the known training group. This kind of classification is also call the supervised learning method [12].

DTs are generally generated from top to bottom. Each event or decision may lead to two or more events, leading to different results. Drawing this decision branch into a graph will result in it looking like a tree. The branches and trunks of the tree are called decision trees. At present, DT is widely used in clinical medical diagnosis of illnesses such as breast cancers and cardiovascular diseases [13]. While falls and balance can be effectively assessed using CoP signals [14], questionnaire measurements are still useful data relied upon by clinical caregivers [15]. However, the measurement of the questionnaire sometimes encounters difficulties in practice due to its physical limitations, which is often an important factor in whether the questionnaire can be collected effectively. CoP data can be beneficial in this situation. Because CoP is highly associated with falls and balance, this study wanted to investigate the possibility that Mini-BESTest scores could be estimated from CoP data with the aid of linear regression or binary classification by regression or a DT. Combined with past understanding of the cutoff point of the surveys, the estimated survey scores can be beneficial for clinical users, thereby improving the care service for individual cases.

## 2. Materials and Methods

### 2.1. Subjects Information and Survey Distributions 

The CoP data used in this study were obtained from a human balance evaluation database provided by the Santos Public Repository and were collected by Santos, D.A., and Duarte, M. [16]. Their study was designed to create and provide a publicly available data set related to human balance for researchers employing quantitative and qualitative evaluations and was approved by the local ethics committee of the Federal University of ABC (#842529/2014). All the data are available at PhysioNet (DOI: 10.13026/C2WW2W) under the ODC Public Domain Dedication and License v1.0 (http://opendatacommons.org/licenses/pddl/1.0/ accessed on 6 April 2021) and at Figshare (DOI: 10.6084/m9.figshare.3394432) under the CC-BY license (https://creativecommons.org/licenses/by/4.0/ accessed on 6 April 2021).

This public database originally recruited 163 subjects, 116 women and 47 men, ranging in age from 18 to 85 years old, with basic data including numbered name, visual status, age, age group (two groups: below and over 60 years old), gender, height, weight, BMI, foot length, nationality, skin color, disease, medication, corrective device or prosthesis, and date of test. In addition to the above basic information, there are other personal questionnaires related to falls and physical activity: the International Physical Activity Questionnaire (IPAQ); the Short FES-I and the respective scores of the 7 items; the Mini-BESTest and the respective scores of 14 items; and F12M, the record of falls within the past 12 months. This experiment uses subjects who are belong to the age group of those older than 60 years old. The data of these subjects are organized in Table 1. According to the subjects’ records of disease and medication, their health statuses are-wide ranged. This phenomenon is also reflected in the Mini-BESTest scores.

### 2.2. CoP Recording and CoP Feature Extraction

The force plate used in this study is an OPT400600-1000 (AMTI; Watertown, NY, USA) and an amplifier (Optima Signal Conditioner; AMTI; Watertown; USA) with a size of 400 × 600 × 82.55 mm, capable of measuring force and moment on the *x*, *y*, and *z* axis (Fx, Fy, Fz, Mx, My, Mz). The sampling frequency is 100 Hz, and the CoP signals are derived from force and moments with an accuracy of 0.2 mm. The subjects were randomly balanced for 60 s each recording period, and there were four different recording conditions: CR with eyes closed on a rigid surface, OR with eyes open on a rigid surface, CF with closed eyes on a foam pad, and OF with eyes opened on the foam pad; each condition was performed three times. Each CoP recording can be derived into two CoP time series, CoPx and CoPy. The following five CoP parameters are derived for each CoP time series: root–mean–square deviation (RMSD), approximate entropy (ApEn), sample entropy (SampEn), power of spectral density (PSD), and median frequency (MF) of spectral density. Symbols of these CoP features and Mini-BESTest scores are organized in Table 2. In addition to CR, OR, CF, and OF, C4 = CR + OR + CF + OF is defined as combination of CoP features of 4 CoP recording conditions to eliminate CoP feature deviations due to recording conditions. Approximate entropy and sample entropy are derived by using the pracma package with the approx_entropy and sample_entropy commands in the R language. Entropy has two built-in parameters: dimension setting = 2 and r = 0.2 × STD. STD is the standard deviation of the input signal. tau = 1 for sample entropy. The CoP spectrum is calculated by the pspectrum command of the psd package in the R language.

### 2.3. Linear Regression

Original Mini-BESTest scores are output as Y, and input are 10 CoP features, defined as X1 to X10.

(1) $$\mathrm{Estimated}Y=a+\sum_{i=1}^{10}a_{i}X_{i},\;I=1\;to\;10.$$

*A_j_* is the regression coefficient of the corresponding input CoP features.

Coefficients can be estimated through minimization of the error between Y and *Estimated Y*. Linear regression was calculated by the *lm* command, and input variable selection was calculated by the *stepAIC* command; both belong to the MASS package in the R language.

### 2.4. Binary Classification Performance Evaluation by Regression and Decision Tree

There are 5 types of input feature vector: C4, CF, OF, CR and OR. Each feature vector contains 10 features derived from CoPx and CoPy. The training group to testing group ratio is 80% to 20%. A DT is used for binary classification. Subjects with higher and lower Mini-BESTest cutoff survey scores are classified. Mini-BESTest scores ranging from 14 points to 26 points are examined. For example, when we deal subjects with the Mini-BESTest at a cutoff score of 20 points, there are two group; one is subjects with more than 20 points, and the other group comprises those with 20 point or below. The DT will produce a decision regarding whether the subject’s survey is higher or lower than 20 points based on the input CoP feature vector. The classification result will have the following four possibilities: be classified as “above the score group” by the DT and be “above the score group” (TP); be classified as “above the score group” by the DT but not “above the score group” (FP); be classified as “less than or equal to the score group” by the DT and as “less than or equal to the score group” (TN); or be classified as “less than or equal to the score group” by the DT but not “less than or equal to the score group” (FN). Classification performance would be further evaluated by accuracy (ACC), defined as follows:

ACC = (TP + TN)/(TP + TN + FP + FN) × 100% (2)

In addition, the receiver operating characteristic (ROC) curve is also calculated. The ROC curve is a coordinate-shaped analysis tool; it is not affected by the extreme values of the training data and testing data and can give an evaluation of classification. In the ROC curve space, we put the FPR (false positive rate) on the *x* axis and the TPR (true positive rate) on the *y* axis. The area under the curve of the ROC (AUC) is to observe the pros and cons of the model. The area will be between 0 and 1. The closer the AUC is to 1, the better the classification effect of the model. Each survey score has a corresponding AUC. The above calculation is implemented in the R language, and the DT is evaluated with the CART package in R.

### 2.5. Statistics

**Figure 1:**
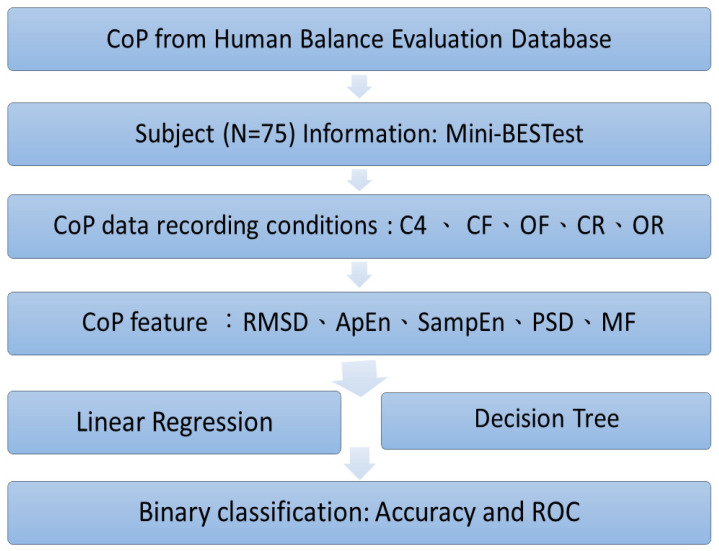
Experiment flowchart.

The means and standard deviations of following features are estimated: subject information, inclusive of their age, height, weight, and corresponding real and estimated Mini-BESTest scores by the linear regression model. Also, the mean and standard deviation of the decision tree classification accuracy are calculated. The complete experimental process is shown in Figure 1.

## 3. Results

**Figure 2:**
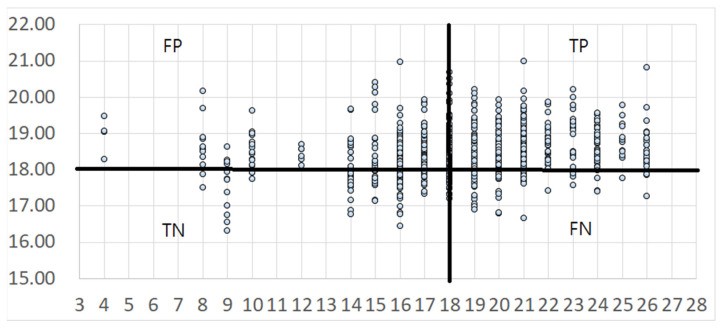
*x*-axis is real Mini-BESTest score; *y*-axis is Mini-BESTest score as estimated by linear regression.

The final regression model is estimated as follows:

*Estimated Y* = 17.593 − 21.146 * X5+ 29.894 * X6+ 3.650 * X10, (3)

where *Estimated Y* is estimated Mini-BESTest score, with an adjusted R-squared value of 0.02. The meanings of X5, X6, and X10 are listed in Table 2. The distribution of real Mini-BESTest scores and estimated Mini-BESTest scores is shown in Figure 2. The estimated Mini-BESTest scores range from 16.7 to 20.8, with the same mean as the real Mini-BESTest scores, and the standard deviation is 0.65. The correlation coefficient between the real and estimated Mini-BESTest scores is 0.18. Apparently, the regression result is not satisfactory.

**Figure 3:**
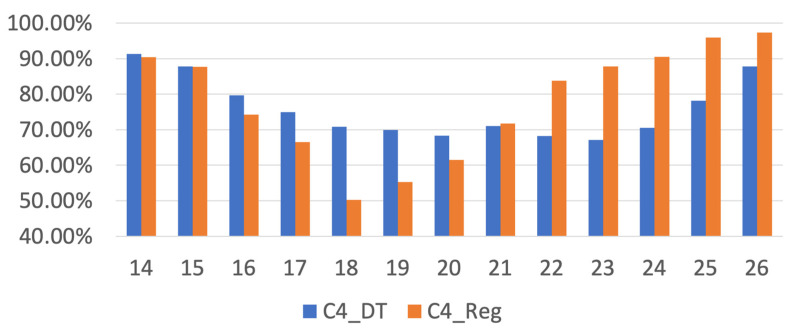
Binary classification accuracy by DT (C4_DT) and linear regression (C4_Reg) for Mini-BESTest. *x*-axis is Mini-BESTest score; *y*-axis is classification accuracy.

**Figure 4:**
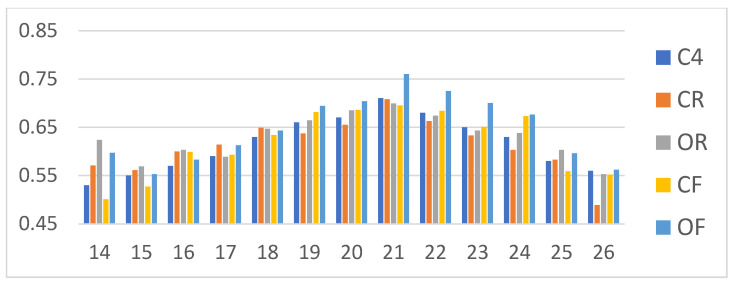
Mini-BESTest score vs. AUC. *x*-axis is Mini-BESTest score; *y*-axis is AUC.

The binary classification results are displayed in Figure 2. The *x*-axis is real Mini-BESTest score, and the *y*-axis is the estimated Mini-BESTest score by the regression model. Suppose we take a cutoff score = 18 as an example. When *x*-axis > 18 and y > 18, it is TP. When *x*-axis > 18 and y ≤ 18, it is FN. When *x*-axis ≤ 18 and y > 18, it is FP, and when *x*-axis ≤ 18 and *y*-axis ≤ 18, it is TN. The binary classification accuracy then can be derived according to Equation (2). The binary classification results of Mini-BESTest scores by the DT and linear regression are shown in Figure 3 and Table 3. Among Mini-BESTest scores 16 to 21, the classification accuracy of the DT is higher than that of linear regression. Figure 4 is the classification AUC for different cutoff point scores under different CoP measurement conditions. The results show that the ACC will first decrease and then increase with the increase of the cutoff point, and the AUC follows the opposite trend; it will first increase and then decrease. The minimum ACC is at 20 scores for the OF condition and at 23 scores for the rest of the measurement conditions from 19–26 points. The maximum AUC is at 21 scores for OF. The AUC of OF is higher than that of the other measurement conditions from 19–26 points.

## 4. Discussion

The innovation of this article is to use CoP measuring equipment to estimate the Mini-BESTest score, which has been established for a long time in the field of balance assessment in the elderly. The Mini-BESTest seems to be low cost, and the time spent on it is not large. However, this research method is mainly based on the fact that some elders are unwilling to complete the Mini-BESTest, but they will accept CoP measurements. Although this is not usual for cases in practice, it does happen at times in medical and clinical environments. This study was also undertaken at the request of a clinician’s proposal. The cost of CoP measurement is higher than that of the Mini-BESTest, so this is not a usual case. However, it truly is a clinical need for a small portion of subjects.

The original purpose of this study is to use CoP parameters to estimate the Mini-BESTest score. To solve this problem, traditional linear regression analysis is the preferred method. However, the result of linear regression analysis is not ideal for this topic. We further examined the binary classification performance of linear regression analysis and compare it with that of the DT. Among Mini-BESTest scores from 16 to 21, the DT performed better than linear regression analysis. This score range is also exactly the cutoff score range used by Mini-BESTest to judge clinically important diseases. The Mini-BESTest has a score ranging from 0 to 28 points. There are 14 items in the whole questionnaire, with “0” representing the lowest level of function and “2” representing the highest level of function. If a subject must use an assistive device for an item, the item’s score is lowered by one. The results of this study showed that the best resolution for the Mini-BESTest cutoff point was at 21 points.

In clinical research related to Mini-BESTest cutoff points, such as Anson’s study of an elderly group and another elderly group with a history of falls, the cutoff points of Mini-BESTest scores are 19.0 (3.8) and 19.3 (3.1), respectively [17]. For myotonic dystrophy type 1 subjects, there is a cutoff score of 21.5 to identify fallers [18]. Lopes investigated the Mini-BESTest scores distribution among Parkinson’s disease patients. There are 204 non-fallers, with a score of 20.4 (6.0), and 166 fallers with a score of 16.8 (7.1) [19]. For stroke subjects with age 60.8 (9.4), the Mini-BESTest score was 17.4 (10.6), and it changed to 22.4 (5.2) after a conventional rehabilitation with an average length of 17 weeks [20]. Magnani et al. showed that the cutoff points to distinguish fallers are different for various age groups. The cutoff score was 25 points for people 60 to 69 years of age, 23 points for the age group of 70 to 79 years, 22 points for people 80 to 89 years of age, and 17 points for people 90 years of age or older [21]. Because CoP mainly detects the balance mechanism of the body, and Mini-BESTest is also a test for balance, using CoP to push the results of the Mini-BESTest back and forth can yield good results.

There are severe limitations in our study. This study uses an open database, and the data source will limit the applicability of these research results. According to the conclusion from Duchesne, E., et al. [18], the Mini-BESTest scores corresponding to fallers of different ages are different, but this database contains data from some patients aged 60–86. Because the subjects were not numerous enough to test the Mini-BESTest according to their age groups, they were mixed and tested together. This is also another limitation of this study. In addition, some of the elderly people in this database have a history of falls, but there are not many people with extreme physical disabilities, which can also be seen from the average scores and standard deviations of the Mini-BESTest scores. Therefore, the results mainly provide a potential analysis of using CoP to estimate the questionnaire score, which is also mainly applicable to those elderly without extreme physical abnormalities. In the future, the relation between Mini-BESTest scores and CoP data for clinical subjects, such as stroke patients, with high fall risk is expected. In addition, data from other sensors are also potential sources for Mini-BESTest score estimation, such as inertial measurements [22], gait parameters [23], and EMG [24]. The study proposes a novel model to estimate cutoff survey scores using measurement sensors. This model can be applied to other interesting surveys.

In addition, although this study only uses decision trees as the criterion for assessment, other artificial intelligence algorithms may also improve AUC, but this study is mainly aimed at the classification of cutoff points. Whether other classification algorithms may be superior to decision tree algorithms is still an interesting topic for future study. A decision tree algorithm sees very successful application in this example, providing a clinically reasonable cutoff point classification.

## 5. Conclusions

Some elderly people are more resistant to taking a questionnaire than undergoing a physiological measurement. Therefore, this study uses a 60 s CoP recording to estimate a Mini-BESTest score. To estimate a Mini-BESTest score from CoP data by the traditional regression method is not a good approach because the correlation coefficient is very small. Both linear regression and a DT are compared to estimate the classification accuracy of different cutoff score groups. It is a better approach to classify Mini-BESTest scores by CoP data into binary categories: above and below the cutoff Mini-BESTest score. The binary classification performance is acceptable. Among Mini-BESTest scores from 16 to 21, the DT is superior to linear regression. CoP measurement is faster than the Mini-BESTest. The findings of this study can be used as an auxiliary questionnaire score estimation tool for elderly patients from whom it is not easy to obtain questionnaire data in the future.

## Figures and Tables

**Table 1:** Subject information (N = 75). Data are represented as average (standard deviation).

Subject Numbers	Male 15/Female 60
Age (year)	71.3 (6.5) [max = 85.8, min = 60.5]
Height (cm)	157.3 (8.2) [max = 178.5, min = 140]
Weight (kg)	63.3 (8.3) [max = 75.9, min = 44.2]
Mini-BESTest scores	18.2 (4.0) [max = 26, min = 4]

**Table 2:** Symbols of these CoP features and Mini-BESTest scores used in this study.

Symbol	Corresponding Meaning
Y	Original Mini-BESTest scores
X1	RMSD. CoPx
X2	RMSD. CoPy
X3	ApEn. CoPx
X4	ApEn. CoPy
X5	SampEn. CoPx
X6	SampEn. CoPy
X7	PSD. CoPx
X8	PSD. CoPy
X9	MF. CoPx
X10	MF. CoPy

**Table 3:** Binary classification accuracy of Mini-BESTest cutoff scores by DT and linear regression (_Reg) under four CoP recording conditions.

Mini-BESTest Cutoff Scores	CR_DT	OR_DT	CF_DT	OF_DT	CR_Reg	OR_Reg	CF_Reg	OF_Reg
14	89.74%	91.84%	93.26%	93.80%	88.94%	88.16%	90.94%	91.78%
15	87.24%	86.12%	90.53%	88.13%	86.28%	85.53%	88.22%	89.04%
16	80.66%	81.07%	81.37%	79.95%	75.22%	71.93%	74.62%	75.34%
17	75.46%	74.08%	77.95%	76.77%	64.60%	65.79%	66.47%	67.12%
18	72.50%	71.89%	72.47%	71.98%	51.77%	49.12%	51.06%	51.60%
19	67.91%	71.12%	72.84%	73.28%	56.64%	52.19%	54.38%	55.25%
20	66.84%	70.26%	70.74%	72.08%	61.06%	63.16%	61.63%	62.10%
21	70.82%	69.90%	69.79%	76.15%	72.12%	72.37%	71.60%	71.23%
22	66.38%	67.70%	68.63%	72.50%	84.07%	84.21%	83.69%	83.56%
23	66.33%	66.63%	66.68%	71.61%	88.05%	88.16%	87.76%	87.67%
24	69.64%	71.17%	73.21%	74.17%	90.71%	90.79%	90.48%	90.41%
25	76.84%	78.37%	75.95%	78.80%	96.02%	96.05%	95.92%	95.89%
26	86.33%	87.81%	87.16%	88.49%	97.35%	97.37%	97.28%	97.26%

## Data Availability

The data that support the findings of this study are available from the corresponding author, upon reasonable request.

## Abbreviations

BESTest	Balance Evaluation Systems Test
Mini-BESTest	Mini-Balance Evaluation Systems Test
CoP	center of pressure
DT	decision tree
BMI	body mass index
IPAQ	The International Physical Activity Questionnaire
F12M	fall within 12 months
Short FES-I	Short Falls Efficacy Scale International
RMSD	root–mean–square deviation
ApEn	approximate entropy
SampEn	sample entropy
PSD	power of spectral density
MF	median frequency
STD	standard deviation
TP	true positive
FP	false positive
TN	true negative
FN	false negative
ROC	receiver operating characteristic
AUC	area under the curve
